# The Importance of Cardiorespiratory vs. Muscular Fitness in Reducing the Odds of Hypertension in War Veterans: A Population-Based Study

**DOI:** 10.3390/ijerph182111120

**Published:** 2021-10-22

**Authors:** Mario Kasović, Lovro Štefan, Zvonimir Kalčik

**Affiliations:** 1 Faculty of Kinesiology, Department of General and Applied Kinesiology, University of Zagreb, 10000 Zagreb, Croatia; mario.kasovic@kif.hr; 2 Faculty of Sports Studies, Department of Sport Motorics and Methodology in Kinanthropology, Masaryk University, 62500 Brno, Czech Republic; 3 Faculty of Science, Department of Recruitment and Examination (RECETOX), Masaryk University, 62500 Brno, Czech Republic; 4 Home of Croatian Veterans, 10110 Zagreb, Croatia; zvonimirkalcik@gmail.com

**Keywords:** blood pressure, physical fitness, veterans, relationship

## Abstract

Purpose: The purpose of the study was to examine separate and combined associations between cardiorespiratory fitness (CRF) and musculoskeletal fitness (MF) with hypertension. Methods: In this cross-sectional study, participants were 764 men and women aged 45–75 years, who were part of the Homeland War between 1990 to 1995 (33.5% women). CRF included the 2-min step test, while MF was consisted of push-ups in 30 s, chair-stands in 30 s and sit-ups in 30 s. The prevalence of hypertension was defined according to new American College of Cardiology and American Hearts Association Blood Pressure Guidelines for systolic and diastolic blood pressure of ≥130 mmHg and/or ≥80 mmHg. Results: In models adjusted for sex, age, fatness and fasting blood glucose, we found a graded inverse association between CRF and MF with hypertension. Less cardiorespiratory and muscular fit individuals were more likely to have hypertension. When CRF and MF were combined, individuals with high MF and low CRF, low MF and high CRF and low MF and CRF were 1.77, 2.15 and 7.09 more likely to have of hypertension. Conclusion: Both CRF and MF are associated with the prevalence of hypertension, while the magnitude of the associations between MF and hypertension was more pronounced.

## 1. Introduction

Cardiovascular diseases (CVD) cause the highest number of premature deaths worldwide [1], with hypertension being its leading factor [2,3]. In 2010, 31.3% of the global adult population had hypertension [4]. The prevalence of hypertension changes globally, where low- and middle-income countries had an increasing trend of hypertension over the past two decades [5], while high-income countries experienced a modest decrease over the same period [3]. The transitions in economy, dietary patterns and lifestyle habits have led to unhealthy diets and lack of physical activity (PA) [3], which have been the most significant factors for developing the risk of hypertension [2].

It has been well-documented, that the higher levels of PA have been inversely associated with a lower incidence of chronic diseases and their risk factors [6,7,8], including hypertension. Specifically, the evidence suggests that, in addition to anti-hypertensive therapy, acute exercise has been associated with immediate reductions in systolic blood pressure, regardless of sex, age and ethnicity [9]. More frequent participation in exercise may even result in more pronounced reductions in blood pressure [10]. From the physiological point of view, exercise may have favorable effects on oxidative stress, inflammation, renin-angiotensin system activity and insulin sensitivity [11].

Although health-benefits of PA on hypertension are well-known [6,7,8,9,10,11], the evidence has been provided regarding the associations between various physical fitness components and hypertension [8,12,13,14,15,16,17,18]. A longitudinal study by Carnethon et al. [8] showed that during the 15-year period, participants with low cardiorespiratory fitness (CRF; <20th percentile) were 3- to 6-fold more likely to develop hypertension, compared to the participants with high CRF (≥60th percentile). Another study aimed to examine the associations between CRF, and elevated blood pressure showed that participants in the highest CRF tertile had the lowest blood pressure values [12]. Similar associations have been obtained for musculoskeletal fitness (MF), where higher levels of MF are consistently associated with lower prevalence of hypertension in both longitudinal [16] and cross-sectional studies [17,18]. In addition, the inverse associations between CRF and MF and cardiovascular risk were partially explained by the associations between fitness and fatness [8,12]. However, findings from other studies show the inverse associations between CRF and cardiovascular risk, independent of fatness [19,20].

Compared to the general population, war veterans are at increased risk for developing hypertension [21], having lower levels of physical functioning and quality of life [22]. It has also been reported that veterans describe challenges in maintaining their physical performance [23], being more prone to cigarette smoking [24] and gaining weight [25], which are all independent predictors of hypertension and physical fitness. This specific population often struggles to maintain healthy quality of life and longevity, because of stressful situations during combat, which often leads to posttraumatic stress disorder and other forms of physical and mental dysfunction. Although an effort has been made to help the veterans from Croatia through guided physical activity and psychological councelling [21], they are still an at-risk group for having cardiovascular complications and poor physical performance.

As such, the associations between CRF and MF with hypertension in war veterans remain unknown. By examining the associations between CRF and MF with hypertension, health-related professionals and trainers may be more aware of which aspect of physical fitness is more protective against the prevalence of hypertension, and which types of training modes should be implemented within the rehabilitation centers.

Therefore, the purpose of the study was to examine separate and combined associations between CRF and MF with hypertension. We hypothesized, that both CRF and MF would be inversely associated with hypertension, while the combination of low CRF and MF would have the strongest association with hypertension.

## 2. Materials and Methods

### 2.1. Study Participants

In this cross-sectional study, we recruited men and women aged 45–75 years, who participated in a homeland war between Croatia and Serbia from 1990 to 1995. More detailed information about the rehabilitation institution and specific programs done with war veterans are described elsewhere [21]. In brief, The Home for Croatian Veterans is an accommodation and rehabilitation institution established by the Ministry of Croatian Veterans with the intention of improving and strengthening the provision of comprehensive care for the veteran and suffering population. The general goal of the Home of Croatian Veterans is to preserve the acquis and mitigate the negative consequences of the Homeland War. The special goal refers to strengthening the care system and raising the quality of life of the veteran and suffering population through the activities of the Home of Croatian Veterans. The institution provides the user with the use of accommodation or accommodation services. The accommodation program includes the use of services during the provided accommodation in the Institution 24 h a day for up to 20 days. The program of stay implies the use of the services of the Institution during a full-day or half-day stay. A half-day stay can last up to 6 h a day. The full day stay can last from 6 to 10 h a day. The program of the stay does not include overnight stays in the Institution and is intended for users who live near the Home and use the partial services of the Home. For the purpose of this study, we collected the data from 2017 to 2020 for all of the participants within the facility care. During the period of 4 years, approximately 2500 users used the accommodation service. The inclusion criteria to be part of the study were: (1) being without chronic diseases, (2) having no history of psychiatric symptoms or treatments, (3) being able to perform CRF and MF tests and (4) participating in the Homeland War for at least 100 days. Of 2500, 764 war veterans met the inclusion criteria. By using such a sample size, two-tailed test, α < 0.05, and the statistical power of 0.95, we would be able to detect a minimum effect size of 0.13. Before the study began, all of the participants gave written informed consent for participation. All of the procedures were anonymous and in accordance with the Declaration of Helsinki and were also approved by The Home for Croatian Veterans (Ethics code number: 2017/04).

### 2.2. Blood Pressure Assessment

Blood pressure was measured with a standard mercury sphygmomanometer blood pressure cuff, according to the American Heart Association’s standardized protocol [26], with three times in a sitting position after a 5 min rest period. Specifically, a standard mercury sphygmomanometer was placed on the right mid-arm at the same level as the heart. The average of three measurements was recorded for systolic and diastolic blood pressure. The measurer was not dressed as a doctor during the measurement, an effort to prevent “white-coat hypertension syndrome”. According to new American College of Cardiology and American Hearts Association Blood Pressure Guidelines, the prevalence of hypertension was diagnosed when blood pressure was ≥130 mmHg and/or ≥80 mmHg [27]. Of note, no antihypertensive drugs were used prior to or during the study by the study participants.

### 2.3. CRF

To assess the level of CRF, we administrated the 2-min step test [28]. The procedure for performing the test has been described previously [28]. In brief, the participant stands up straight next to the wall while a mark is placed on the wall at the level corresponding to midway between the patella (kneecap) and iliac crest (top of the hip bone). When the measurer gives the signal, the participant starts to march in place for two minutes, lifting the knees to the height of the mark on the wall. If the participant needs to rest, it is allowed by permitting them to hold onto the wall or a stable chair. The test is completed after two minutes of stepping and the result is expressed as the total number of times the right and left knees reach the tape level in two minutes. Previous evidence confirms an excellent test-retest reliability property (ICC = 0.90, *p* < 0.001) and satisfactory validity, when compared with treadmill performance (*r* = 0.74, *p* < 0.001) [28]. Also, the 2-min step test may successfully detect expected declines across different age groups and significant differences in active vs. inactive individuals [28].

### 2.4. MF

To assess muscular dynamic endurance and an ability to stabilize the upper body, the push-up test in 30 s was applied [29]. Alternately, if the participants could not complete the push-up test, the test was carried out on the knees [29]. The final score was recorded as the number of push-ups in 30 s.

The chair stands in 30 s is the test developed to assess lower body strength [28]. It consists of standing up and sitting down from a chair as many times as possible within 30 s. The participant sat on the standard chair (with an approximate height of 40 cm) with their back in an upright position. Each participant was instructed to look straight ahead and to rise with their legs fully extended after the “1, 2, 3, go” command at their own preferred speed and with their arms folded across their chest. All trials were performed using the same chair and with similar ambient conditions [30]. The final score involved counting the number of stand-ups in 30 s.

The sit-up test was performed to assess repetitive upper body strength [31]. The participants were instructed to perform as many correct bent-knee sit-ups as possible in 30 s, while lying on a mat in a supine position with the knees bent at an angle of approximately 90° and keeping the feet together [31]. Arms were placed on the chest with the hands-on opposite shoulders. From the initial position, each participant performed a full sit-up to the upright position with their elbows touching their thighs and then returned to the supine position where their shoulders (scapula) touched the mat surface [31]. The performance was scored as having as many correct sit-ups as possible in 30 s.

### 2.5. Fat Mass Assessment

To assess body composition, we used bioelectrical impedance analysis (Omron BF500 Body Composition Monitor, Omron Medizintechnik, Hamburg, Germany). The device uses eight electrodes and requires the participant to stand on metal footpads in bare feet and grasp a pair of electrodes fixed on a handle with arms extended in front of the chest [32]. The manufacturer’s pre-programmed equations were used to predict fat mass. All participants were instructed not to consume food or water before the testing. The same equipment was used for each participant. Standing height and weight were measured following the instruction from previous studies [33] by using Seca portable 202 scales (Seca, Hamburg, Germany) and a digital scale (Seca, model 769).

### 2.6. Fasting Blood Glucose

Blood samples were collected in the morning hours in resting seated position after a 12-h overnight fast. Each blood sample was drawn from the forearm using a vacutainer blood collection tube with a needle. Laboratory measurement of fasting blood glucose was performed according to standardized procedures.

### 2.7. Data Analysis

Basic descriptive statistics are presented as mean and standard deviation (SD). Sex differences in anthropometric, blood pressure, fasting blood glucose, CRF and MF were examined using the analysis of covariance (ANCOVA), adjusted for age for normally and Man-Whitney test for not normally distributed variables. Cohen *d* effect sizes (ES) were calculated to determine the magnitude of the sex differences. ES was classified as trivial (<0.2), small (0.2–0.6), moderate (0.6–1.2), large (1.2–2.0), very large (>2.0) and extremely large (>4.0) [34]. To examine the test-retest reliability administrated three weeks apart (21 days), we used the intraclass correlation coefficient (ICC) and Cronbach’s α. Data from CRF and MF tests were used as continuous variables and converted into quartiles. The most fit individuals were categorized in the fourth quartile and were served as a reference category in all calculations. The outcome measure was the presence of hypertension (≥130 mmHg and/or ≥80 mmHg). The associations between CRF and MF with hypertension were examined using Generalized Linear Models (GLMs) with binary logistic regression analyses. Odds ratios (OR) and 95% confidence intervals (95% CI) were used to present the presence of hypertension. CRF and MF tests were separately entered into each model. Sex, age, fatness and fasting blood glucose were used as covariates in adjusted models. Data from three MF tests were *z*-transformed, by summing an individual push-up, chair-stand and sit-up *z*-scores. To examine the combined associations of CRF and MF with hypertension, we classified the participants as having low or high CRF and MF, based on median splits [35]. Therefore, four categories were created, as follows: (1) high MF and high CRF, (2) high MF and low CRF, (3) low MF and high CRF and (4) low MF and low CRF. The highest muscular and cardiorespiratory fit individuals in binary logistic regression analyses served as the reference category for the prevalence of hypertension. All analyses were performed in Statistical Packages for Social Sciences (SPSS) version 23 (SPSS Inc., Chicago, IL, USA).

## 3. Results

Basic descriptive statistics of the study participants are presented in Table 1. Men were taller, heavier, and had less fat mass %, compared to women. Women had smaller values in systolic and diastolic blood pressures, compared to men. Men achieved better values in MF, while no significant differences in CRF between sexes were observed.

Table 2 shows test-retest reliability data for MF and CRF with three weeks’ interval (21 days) between duplicate tests. No significant differences between duplicate tests were found (*p* > 0.05). The battery of tests had good to excellent reliability and concordance of the ICC between the two trials.

In unadjusted models, the univariate binary logistic regression showed that the prevalence of hypertension linearly increased with decreasing ‘push-ups in 30 s’: Q4–OR = 1.00 (reference), Q3–OR = 1.87 (95% CI 1.17 to 3.01, *p* = 0.010), Q2–OR = 3.49 (95% CI 2.11 to 5.77, *p* < 0.001) and Q1–OR = 9.60 (95% CI 5.43 to 16.95, *p* < 0.001); ‘chair-stands in 30 s’: Q4–OR = 1.00 (reference), Q3–OR = 3.49 (95% CI 2.00 to 6.08, *p* < 0.001), Q2–OR = 4.55 (95% CI 2.69 to 7.69, *p* < 0.001) and Q1–OR = 6.29 (95% CI 3.65 to 10.83, *p* < 0.001); ‘sit-ups in 30 s’: Q4–OR = 1.00 (reference), Q3–OR = 3.39 (95% CI 2.08 to 5.53, *p* < 0.001), Q2–OR = 3.99 (95% CI 2.35 to 6.78, *p* < 0.001) and Q1–OR = 9.97 (95% CI 5.54 to 17.95, *p* < 0.001); and ‘2-min step test’: Q4–OR = 1.00 (reference), Q3–OR = 1.89 (95% CI 1.20 to 2.99, *p* = 0.006), Q2–OR = 2.46 (95% CI 1.57 to 3.86, *p* < 0.001) and Q1–OR = 3.10 (95% CI 1.97 to 4.86, *p* < 0.001). Compared to the most muscular and cardiorespiratory fit individuals (high MF and CRF), those categorized as having high MF and low CRF, low MF and high CRF and low MF and CRF were 2.03 (95% CI 1.12 to 3.45, *p* = 0.009), 3.61 (95% CI 2.06 to 6.33, *p* < 0.001) and 15.60 (95% CI 7.85 to 40.00, *p* < 0.001) more likely for the presence of hypertension.

Table 3 shows the adjusted associations between MF and CRF with the prevalence of hypertension. After the models were adjusted for sex, age, fatness, and fasting blood glucose, the magnitude of the associations between MF and CRF with the prevalence of hypertension was notably reduced. However, less musculoskeletal and cardiorespiratory fit individuals remained having higher odds for the prevalence of hypertension, compared to the most fit individuals. When MF and CRF were inserted simultaneously into the model (MF*CRF), individuals with high MF and low CRF, low MF and high CRF and both low MF and CRF had 1.77, 2.15 and 7.09 more odds for the prevalence of hypertension.

## 4. Discussion

The main purpose of the study was to examine cross-stional associations between CRF and MF with hypertension in a large sample of war veterans. Our main findings are: (1) lower levels of MF and CRF are significantly associated with the presence of hypertension, (2) after adjusting for sex, age, fatness and fasting blood glucose, lower levels of MF and CRF remain significantly associated with the presence of hypertension and (3) participants with high MF and low CRF, low MF and high CRF and low MF and CRF have higher odds for the presence of hypertension, compared to the most fit individuals.

The prevalence of hypertension in war veterans is still unknown. It is often associated with having other co-morbidities, including older age, higher body-mass index, cerebrovascular attack, peripheral vascular disease, congestive heart failure, chronic kidney disease, diabetes mellitus and metabolic syndrome [36].

Our findings suggest that CRF is inversely associated with the presence of hypertension, which is in line with previous longitudinal [8,13,14,15] and cross-sectional studies [12]. Specifically, a longitudinal study by Carnethon et al. [8] showed that during the 15-year period, participants categorized as having low CRF (<20th percentile) were 19% more likely to develop hypertension. Other studies found similar findings, where with each 1-metabolic equivalent increment in treadmill performance, the incidence of hypertension decreased by 19% [14,15]. In addition, the same study reported that the incidence for hypertension decreased by 65% in highly fit women [15]. Higher level of CRF may be an important homeostatic factor that significantly contributes to the regulation and maintenance of healthy blood pressure [15]. Moreover, it has been suggested that improving CRF throughout moderate-intensity PA may have beneficial effects on hemodynamics and cardiac performance, improving function of the left ventricle [37].

Our findings also suggest that various MF tests are significantly associated with hypertension. Indeed, the majority of studies that have examined the associations between physical fitness and health-related outcomes have predominantly used CRF as the proxy of physical fitness [38]. Similar to CRF, both longitudinal [16] and cross-sectional studies [17,18] have shown that higher levels of MF may reduce the level of hypertension. During a mean follow-up of 19 years, a study by Maslow et al. [16] showed a significant association between muscular strength and incidence of hypertension in baseline prehypertensive men. In addition, the same study reported that CRF was a stronger predictor for incidence of hypertension, compared to MF; that is, a higher level of MF in each CRF level could only provide small additive protective associations against developing hypertension [16]. Such findings are somewhat contradictory, compared to this study. Differences may be explained by using different CRF and MF tests; Maslow et al. [16] assessed the level of CRF and MF by using a maximal treadmill test and one-repetition maximum supine bench press and leg press, while the 2-min step test was used as a proxy of CRF and *z*-transformed scores in push-ups, chair-stands and sit-ups in this study. Although the 2-min step test shows good validity properties [25], the treadmill test is a more objective method, often used as the gold standard for assessing CRF. Moreover, the aforementioned study included only men with a wider age span (20–82 years), and the physical fitness of the participants was categorized into tertiles [16]. One cross-sectional study confirmed the findings from our study, where MF assessed by isometric handgrip strength was significantly associated with hypertension [17].

Finally, we found a significant and strong inverse association between the combined effects of CRF and MF and the presence of hypertension. Compared with those with both high MF and CRF, having high MF and low CRF showed smaller OR (1.77), while the opposite (having low MF and high CRF) was more strongly associated with increased odds of hypertension (OR = 2.15). This indicates that MF may be more important in decreasing the odds of hypertension than CRF. According to previous evidence, CRF is more protective against the odds of hypertension [8,12,13,14,15], compared to MF, which is not in line with our findings. One reason for our finding could be a difference in the way CRF and MF were assessed. Namely, CRF was measured indirectly, while previous evidence used direct methods to assess CRF. It has been highlighted, that the 2-min step test has acceptable validity and only shares ≈50% of the variance with the treadmill protocol [25]. In addition, MF was composed of push-ups, chair-stands and sit-ups, which covered muscular strength and muscular endurance of the whole body. Therefore, the composite score of MF may be more complete, opposed to CRF assessed by only one test. Still, the fact that both low CRF and MF increased the odds of hypertension supports previous evidence that the combinations of aerobic and muscle-strengthening activities should be provided for preventing cardiovascular diseases [39].

This study has a few strengths. First, the findings of the study were based on a large representative sample of war veterans aged 45–75 years. Second, several different MF tests were applied, to create a composite *z*-score for overall MF. Third, the associations between CRF and MF with hypertension were additionally adjusted for sex, age, fatness (fat mass using bioelectrical impedance) and fasting blood glucose.

However, this study is not without limitations. By using a cross-sectional design, we cannot establish the causality of the association, where the prevalence of hypertension might have led to lower levels of CRF and MF. Next, CRF was assessed by the 2-min step test, which was only moderately correlated with the treadmill protocol and underestimated the level of CRF. Finally, we did not adjust for smoking habits and other physiological (for example heart rate) and biochemical parameters, which have been previously associated with hypertension [24] and physical fitness [22,23]. Future research should focus in exploring longitudinal associations between CRF and MF using more objective methods, to determine the causality and different dependent and independent effects of CRF and MF having on the prevalence of hypertension.

## 5. Conclusions

We found inverse associations between CRF and MF with the prevalence of hypertension; that is, lower levels of CRF and MF increased the odds of hypertension, independent of sex, age, fatness and fasting blood glucose. MF exhibited a strong protective effect against the prevalence of hypertension, while the effect of CRF, although significant, was less pronounced. Finally, participants categorized as having low both CRF and MF were 7-fold more likely for having hypertension, compared to the most fit individuals (high CRF and MF). Therefore, we suggest that both CRF and MF should be part of the training protocol for decreasing the odds of hypertension in war veterans, confirming the strong validity properties of both CRF and MF tests with health-related outcome.

## Figures and Tables

**Table 1:** Basic descriptive statistics of the study participants (*N* = 764).

	Total (*N* = 764)	Men (*N* = 508)	Women (*N* = 256)	Cohen’s *D*	*p*–Value ^†^
	Mean ± SD	mean ± SD	mean ± SD		
Age (years)	59.9 ± 7.6	60.0 ± 7.8	59.9 ± 7.1	0.01	0.878
Stature (cm)	172.5 ± 9.1	177.1 ± 6.7	163.5 ± 5.9	2.03	<0.001
Body mass (kg)	90.3 ± 18.5	96.2 ± 22.2	78.3 ± 14.7	0.81	<0.001
Body-mass index (kg/m^2^)	29.7 ± 5.5	29.8 ± 5.9	29.4 ± 5.0	0.07	0.303
Fat mass (%)	27.7 ± 12.9	23.9 ± 11.0	35.0 ± 13.3	1.01	<0.001
Systolic blood pressure (mmHg)	130.4 ± 14.3	131.9 ± 14.7	127.4 ± 13.0	0.31	<0.001
Diastolic blood pressure (mmHg)	83.2 ± 8.9	83.9 ± 9.0	81.9 ± 8.7	0.22	<0.001
Fasting glucose (mmol/L)	5.8 ± 1.3	5.8 ± 1.3	5.9 ± 1.3	0.08	0.610
Push-ups in 30 s (reps)	9.7 ± 4.3	10.2 ± 4.9	8.7 ± 3.9	0.31	0.008
Chair-stands in 30 s (reps)	11.4 ± 4.8	12.0 ± 5.1	10.1 ± 3.9	0.37	<0.001
Sit-ups in 30 s (reps)	9.4 ± 3.2	9.9 ± 3.3	8.4 ± 3.0	0.45	0.006
2-min step test (reps)	111.6 ± 19.7	111.4 ± 20.1	112.0 ± 19.0	0.03	0.722

^†^ denotes using the analysis of covariance (ANCOVA) for normally distributed or Man–Whitney *Z*–test for not normally distributed variables; *p* < 0.05.

**Table 2:** Test-retest reliability of health-related physical fitness tests (*N* = 764).

	Mean ± SD	Cronbach’s α	ICC (95% CI)	*p*-Value
Push-ups in 30 s (reps)				
Initial	9.7 ± 6.3			
Final	12.1 ± 7.4	0.953	0.920	<0.001
Chair-stands in 30 s (reps)				
Initial	11.4 ± 4.8			
Final	13.8 ± 5.9	0.935	0.883	<0.001
Sit-ups in 30 s (reps)				
Initial	9.4 ± 5.9			
Final	11.2 ± 6.6	0.917	0.854	<0.001
2-min step test (reps)				
Initial	111.6 ± 19.7			
Final	111.8 ± 18.2	0.875	0.779	<0.001

ICC-intraclass correlation coefficient; 95% CI-95 percent confident interval; *p* < 0.05.

**Table 3:** ORs for the prevalence of hypertension in the study participants (*N* = 764).

	Quartile	Median	OR	95% CI	Wald Statistics	*p*-Value
Push-ups in 30 s (reps) ^†^	4 (ref)	>13.50	1.00			
	3	10.51–13.50	1.31	0.78 to 2.19	1.05	0.307
	2	6.00–10.50	2.73	1.57 to 4.74	12.67	<0.001
	1	<6.00	4.48	2.36 to 8.50	21.09	<0.001
Chair-stands in 30 s (reps) ^†^	4 (ref)	>13.50	1.00			
	3	12.01–13.50	2.93	1.63 to 5.28	12.90	<0.001
	2	9.00–12.00	3.67	2.05 to 6.59	18.97	<0.001
	1	<9.00	3.94	2.12 to 7.30	18.93	<0.001
Sit-ups in 30 s (reps) ^†^	4 (ref)	>12.00	1.00			
	3	10.51–12.00	3.54	2.09 to 5.99	22.23	<0.001
	2	6.75–10.50	3.46	1.95 to 6.16	17.83	<0.001
	1	<6.75	5.40	2.80 to 10.44	25.26	<0.001
2-min step test (reps) ^†^	4 (ref)	>125.00	1.00			
	3	101.01–125.00	2.00	1.23 to 3.26	7.73	0.005
	2	97.00–101.00	2.23	1.38 to 3.61	10.71	<0.001
	1	<97.00	2.37	1.45 to 3.86	11.94	<0.001
MF*CRF	High/high (ref) ^‡^	/	1.00			
	High/low	/	1.77	1.00 to 3.12	3.88	0.049
	Low/high	/	2.15	1.14 to 4.06	5.58	0.018
	Low/low	/	7.09	3.40 to 14.80	27.27	<0.001

^†^ Each model is adjusted for sex, age, fatness and fasting blood glucose; Quartile 4 denotes the most fit individuals; ^‡^ High/high–high MF and CRF; High/low–high MF and low CRF; Low/high-low MF and high CRF; Low/low–low MF and CRF; *p* < 0.05.

## Data Availability

All the data are freely available upon reasonable request from the corresponding author.

## Abbreviations

CRF	Cardiorespiratory fitness
CVD	Cardiovascular diseases
ES	Effect size
ICC	Intraclass correlation
MF	Musculoskeletal fitness
OR	Odd ratio
PA	Physical activity
Q	Quartile
r	Pearson’s coefficient of correlation
β	Beta coefficient
95% CI	95 percent confidence interval
